# TMRS: an algorithm for computing the time to the most recent substitution event from a multiple alignment column

**DOI:** 10.1186/s13015-019-0158-3

**Published:** 2019-11-18

**Authors:** Hisanori Kiryu, Yuto Ichikawa, Yasuhiro Kojima

**Affiliations:** 1 Department of Computational Biology and Medical Sciences, GSFS, University of Tokyo, 5-1-5 Kashiwanoha, Kashiwa, Chiba Japan; 2 Works Applications Co., Ltd., 1-12-32, Akasaka, Minato-ku, Tokyo Japan

**Keywords:** Phylogenetic trees, Comparative genomics, Probabilistic models

## Abstract

**Background:**

As the number of sequenced genomes grows, researchers have access to an increasingly rich source for discovering detailed evolutionary information. However, the computational technologies for inferring biologically important evolutionary events are not sufficiently developed.

**Results:**

We present algorithms to estimate the evolutionary time ($t_{\text {MRS}}$) to the most recent substitution event from a multiple alignment column by using a probabilistic model of sequence evolution. As the confidence in estimated $t_{\text {MRS}}$ values varies depending on gap fractions and nucleotide patterns of alignment columns, we also compute the standard deviation $\sigma$ of $t_{\text {MRS}}$ by using a dynamic programming algorithm. We identified a number of human genomic sites at which the last substitutions occurred between two speciation events in the human lineage with confidence. A large fraction of such sites have substitutions that occurred between the concestor nodes of Hominoidea and Euarchontoglires. We investigated the correlation between tissue-specific transcribed enhancers and the distribution of the sites with specific substitution time intervals, and found that brain-specific transcribed enhancers are threefold enriched in the density of substitutions in the human lineage relative to expectations.

**Conclusions:**

We have presented algorithms to estimate the evolutionary time ($t_{\text {MRS}}$) to the most recent substitution event from a multiple alignment column by using a probabilistic model of sequence evolution. Our algorithms will be useful for Evo-Devo studies, as they facilitate screening potential genomic sites that have played an important role in the acquisition of unique biological features by target species.

**Electronic supplementary material:**

The online version of this article (10.1186/s13015-019-0158-3) contains supplementary material, which is available to authorized users.

## Background

As sequenced genomes continue to accumulate, a very rich source for discovering detailed evolutionary information grows. The UCSC genome browser provides multiple genome alignments for 100 vertebrate species, including humans (the multiz100way track) [1–3].

In previous decades, multiple DNA alignments are often used to reconstruct species trees and ancestral nucleotide states [4] and many algorithms and softwares are developed for such purposes. Some of the most used algorithms include Neighbor-Joining algorithm [5] and maximal likelihood method [4] and Bayesian Markov chain Monte Carlo method [6]. These algorithms usually assume evolutionary models that each nucleotide stochastically mutates over evolutionary time, and output the most consistent phylogenetic tree from possible $(2n-3)!!$ rooted or $(2n-5)!!$ unrooted trees for *n*-species. On the other hand, since the species tree of 100 vertebrates of multiz100way are basically resolved from the previous studies [7], finding functional genomic sites rather than determining the phylogenetic tree is becoming more important application as the use of multiple genomic alignments in recent years.

As it is difficult to visually inspect functional regions from 100-species alignments, computing genome-wide summary statistics is very important. Measuring the strength of negative or positive selection is among the most popular analyses for screening functional regions of genomes [8–14]. These statistics are computed using probabilistic models that model the stochastic processes of DNA mutations along phylogenetic species trees, which are used in tree reconstruction [4–6], and detect genomic regions that show smaller or larger mutation rates using likelihood ratio tests or similar probabilistic computations.

Such statistics have advantages over simpler statistics that do not assume a particular evolutionary model, such as nucleotide frequency of alignment columns and pairwise mismatch rates. By using a phylogenetic tree, we can appropriately count the number of ancestral mutations that are widespread within extant species. Further, stochastic processes can account for multiple nucleotide mutations whose effects are not negligible when we study evolutionarily distant species. However, only conservation/divergence measures are not sufficient to extract all evolutionarily important events from potential $4^{100}$ nucleotide patterns of a 100-species alignment column.

**Fig. 1:**
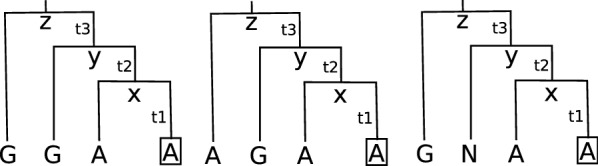
Time to most recent substitution $t_\text{MRS }$. These schematically show the situations that may impact the confidence levels of inferred $t_{\text {MRS}}$ values. The leaf nodes correspond to the target species are indicated by rectangles. In the left figure, we expect the last substitution occurred between node *x* and *y*, and $t_{\text {MRS}}$ will be around $t_1$ to $t_1+t_2$. In the middle figure, the pattern of alignment column is not simple, and the state of node *y* can be either A or G. Therefore, the inferred $t_{\text {MRS}}$ will have a large variance between $t_1$ to $t_1+t_2+t_3$. In the right figure, there is an ambiguous nucleotide in the column. In such cases, the inferred $t_{\text {MRS}}$ value is the same as that inferred from only three species, and the confidence will accordingly be lower than when all four nucleotides are known

In this study, we develop algorithms to compute three statistics, $t_{\text {MRS}}$, $\sigma$, and *q*, for each column of a multiple genome alignment based on an evolutionary model that is similar to those described above. $t_{\text {MRS}}$ is the evolutionary time to the most recent substitution event that occurred along the lineage of a given target species in the phylogenetic tree. Since the confidence in estimated $t_{\text {MRS}}$ values varies markedly among alignment columns depending on gap fractions and complexity of nucleotide patterns (see Fig. 1 for explanation), we also compute the standard deviation $\sigma$ of $t_{\text {MRS}}$. Further, we compute the probability *q* that there is no mutation in the target lineage because the estimated $t_{\text {MRS}}$ value has no meaning in such cases. By filtering out sites with non negligible probability of nucleotide conservation over the entire target lineage based on *q*, we can remove highly conserved sites. By comparing $t_{\text {MRS}}$ with speciation time points, we can categorize sites by the groups of species that share mutation effects with the target species. Such detailed information is difficult to obtain from conservation measures. Our algorithms can be a very useful tool for screening the genomic sites that may have been involved in the acquisition of unique biological features by target species.

In the next section, we describe our algorithms to compute $t_{\text {MRS}}$ and data processing procedures. We first explain the $t_{\text {MRS}}$ algorithm on a single edge of phylogenetic tree, and then generalize it to account for the entire tree. The algorithms for computing $\sigma$ and *q* are described in Additional file 1 as they are very similar to that of $t_{\text {MRS}}$. In the result section, we empirically show the correctness of our algorithms by posterior sampling of mutation history. We also show that our algorithm is fast enough to be applied to the entire human genome, and that $t_{\text {MRS}}$ statistic is very different from other statistics to detect evolutionary conservation/divergence of genomic sites. We then apply our algorithms to the multiz100way dataset and investigate distributions of $t_{\text {MRS}}$ in different genomic contexts. In particular, we investigate the correlation between $t_{\text {MRS}}$ distribution on the bidirectionally transcribed enhancers and tissue specificity of enhancer activities and found that brain-specific transcribed enhancers are threefold enriched in the density of $t_{\text {MRS}}$ that located in the human lineage.

## Method

We first derive formulas for $t_{\text {MRS}}$ and other variables for an edge of a phylogenetic tree, and then describe how to generalize them into statistics for the entire phylogenetic tree.

### Single edge case

A continuous-time Markov model for nucleotide sequence evolution can be defined by a differential equation that determines the time evolution of the probability of observing each nucleotide:

$$\frac{\partial}{{\partial t}}p (a\left| {b,t} \right.) = \sum\limits_{i \in {\text{Nuc}}} {{R_{ai}}} p(i\left| {b,t} \right.),p(a\left| {b,0} \right.) = {\delta _{ab}},$$

where *p*(*a*|*b*, *t*) represents the probability of observing base *a* at time *t* conditioned on base *b* being observed at time zero; $\text {Nuc}=\{A,C,G,T\}=\{1,2,3,4\}$ represents the set of nucleotides; $\delta _{ij}$ represents the Kronecker delta, which is 1 if $i=j$ and is 0 otherwise; and $R=\{R_{ij}\}$ represents the substitution rate matrix. The solution is given by a matrix exponential, which can be numerically computed by using the eigenvalue decomposition of rate matrix $R=U\Lambda U^{-1}$ ($\Lambda =\text {diag}(\lambda _1,\ldots ,\lambda _4)$) as follows [15],

$$\begin{aligned} p(a\left| {b,t} \right.) &=\left[ \exp (tR)\right] _{ab}, \exp (A)=1+\frac{A}{1!}+\frac{A^2}{2!}+\cdots \\&=U e^{t\Lambda } U^{-1}, e^{t\Lambda }=\text {diag}(e^{t\lambda _1},\ldots ,e^{t\lambda _4}) \end{aligned}$$

Similar to the scalar exponential function, a matrix exponential has an infinite product representation,

$$\begin{aligned} \left[ \exp (tR)\right] _{ab}&=\lim _{N\rightarrow \infty }\left[ Q^N\right] _{ab}\\&=\lim _{N\rightarrow \infty }\sum _{X\in \Omega _N(a,b)}Q_{X_NX_{N-1}}\cdots Q_{X_1X_0}, \end{aligned}$$

where $Q=(I+tR/N)$. The matrix *Q* satisfies the condition of a transition matrix of a discrete Markov process for sufficiently large *N*, and our formula for $t_{\text {MRS}}$ can be derived via this connection to the discrete model. In the second equation, $\Omega _N(a,b)$ is the set of all paths *X* along discrete time points $0,\ldots ,N$ such that $X=\{X_k\in \text {Nuc}|k=0,\ldots ,N,X_N=a,X_0=b\}$. Then, the summand of the second equation can be interpreted as the probability of substitution history ${\mathbb {P}}(X_N,X_{N-1},\dots ,X_1|X_0)$. In the discrete model, the random variable $T_{\text {MRS}}$ that represents the time to the most recent substitution is given by

$$\begin{aligned} T_{\text {MRS}}&=\sum _{l=1}^{N-1}\frac{lt}{N}{\mathbb{I}} \left(X_{N}=\cdots =X_{N-l}\right)\mathbb{I}\left(X_{N-l}\ne X_{N-l-1}\right)\\&\quad + \frac{Nt}{N} \mathbb{I} \left(X_{N}=\cdots =X_{0}\right) \\ &= \sum _{l=1}^{N}\frac{lt}{N}{\mathbb{I}}(X_{N}=\cdots =X_{N-l})\\&\quad - \sum _{l=1}^{N-1}\frac{lt}{N}{\mathbb{I}}\left(X_{N}=\cdots =X_{N-l-1}\right) \\&= \frac{t}{N}\sum _{l=1}^{N}{\mathbb{I}}\left(X_{N}=\cdots =X_{N-l}\right), \end{aligned}$$

where ${\mathbb{I}}(\cdot )$ is the indicator function. Note that in the first equation, we define $T_{\text {MRS}}=t$ if path *X* has no substitution at all. In the second line, we used ${\mathbb{I}}(a\ne b)=1-{\mathbb{I}}(a=b)$, and the two terms in the second line mostly cancel out to give the third line. Then, the expected value $t_{\text {MRS}}$ of $T_{\text {MRS}}$ is given by

$$\begin{aligned} t_{\text {MRS}}(a,b,t)&={\mathbb {E}}\left( T_{\text {MRS}}|a,b,t\right)\\& =\sum _{l=1}^{N}\frac{t}{N}{\mathbb {P}}(X_N=\cdots =X_{N-l}|a, b, t) \\&=\left. \sum _{l=1}^{N}\frac{t}{N}\left[ Q_D^{l}Q^{N-l}\right] _{ab}\big /\left[ Q^N\right] _{ab}\right. , \end{aligned}$$

where $Q_D$ is the diagonal part of *Q*.

In order to take the continuum limit ($N\rightarrow \infty$), we use formulas such as

$$\begin{aligned} \sum _{l=1}^{N}\frac{1}{N}f\left( \frac{l}{N}\right)&\rightarrow \int _{0}^{1}f(s)ds \\ Q_D^{l}, Q^{N-l}&\rightarrow \exp (stR_D),\exp ((1-s)tR), (s=l/N), \end{aligned}$$

where $R_D$ is the diagonal part of rate matrix *R*. By using these formulas, $t_{\text {MRS}}$ can be computed using the following formulas

1 $$\begin{aligned} t_{\text {MRS}}(a,b,t)&=\frac{t}{{\mathcal {Z}}}\left[ \int _{0}^{1}e^{stR_D}e^{(1-s)tR}ds\right] _{ab}\nonumber \\&=\frac{t}{{\mathcal {Z}}}\sum _{i=1}^{4} U_{ai}{U^{-1}}_{ib}{\mathcal {K}}(tR_{Daa},t\lambda _i)\nonumber \\ {\mathcal {Z}}&= \left[ e^{tR}\right] _{ab} = \sum _{i=1}^{4} U_{ai}e^{t\lambda _i}{U^{-1}}_{ib}\nonumber \\ {\mathcal {K}}(x, y)&=\int _{0}^{1}e^{sx}e^{(1-s)y}ds= {\left\{ \begin{array}{ll} \frac{e^x-e^y}{x - y} & \quad \text {if } x\ne y \\ e^x & \quad \text {if } x=y \end{array}\right. }, \end{aligned}$$

where $R=U\Lambda U^{-1}$ and $\Lambda =\text {diag}(\lambda _1,\ldots ,\lambda _4)$ is an eigenvalue decomposition of rate matrix *R*. The formulas for the standard deviation $\sigma$ of $T_{\text {MRS}}$ and probability *q* of no substitution can be derived in similar manners and given by

$$\begin{aligned} \sigma (a,b,t)&=\sqrt{{\mathbb{E}}\left( T_{\text{MRS}}^2|a,b,t\right) - t_{\text{MRS}}^2(a,b,t)} \\ {\mathbb {E}}\left( T_{\text{MRS}}^2|a,b,t\right)&=\frac{2t}{{\mathcal{Z}}}\sum _{i=1}^{4} U_{ai}{U^{-1}}_{ib}\mathcal {K'}(tR_{Daa},t\lambda _i), {\mathcal{K'}}(x, y)=\frac{\partial {\mathcal{K}}(x, y)}{\partial x}\\ q(a,b,t)&=\frac{1}{{\mathcal{Z}}}e^{tR_{Daa}}\delta_{ab}. \end{aligned}$$

The derivation of each above formula is described in Additional file 1.

### Strand symmetric rate matrix

Let $a_c$ be the complementary nucleotide of nucleotide *a*. A rate matrix *R* is strand symmetric if it satisfies $R_{a_cb_c}=R_{ab}$ for all $a,b\in \text {Nuc}$ [16]. Strand non-symmetric rate matrices such as the general time reversible (GTR) model generally produce different posterior expectation values if we take the complement of an alignment column. Since there is no specific strand direction in intergenic regions and the existence of two different expectation values for a single genomic site complicates the downstream analyses, we use the most general, 6-parameter strand symmetric rate matrix. Table 1 shows the parametrization of rate matrices of strand symmetric model and GTR model. The parameters are optimized together with the edge lengths of the phylogenetic tree using the maximum likelihood method. We optimize the parameters using a LBFGSB gradient descent package [17], where we compute the gradient of likelihood function exactly using a inside-outside algorithms as described in Refs. [4, 18, 19].

**Table 1:** Rate parameters

$$R_{\text {Symmetric}} = \begin{pmatrix} * &{} \alpha &{} \beta &{} \gamma \\ \eta &{} * &{} \delta &{} \epsilon \\ \epsilon &{} \delta &{} * &{} \eta \\ \gamma &{} \beta &{} \alpha &{} * \end{pmatrix}, R_{\text {GTR}}= \begin{pmatrix} * &{} \pi _A \alpha &{} \pi _A \beta &{} \pi _A \gamma \\ \pi _C \alpha &{} * &{} \pi _C \delta &{} \pi _C \epsilon \\ \pi _G \beta &{} \pi _G \delta &{} * &{} \pi _G \eta \\ \pi _T \gamma &{} \pi _T \epsilon &{} \pi _T \eta &{} * \end{pmatrix}$$	

$R_{\text {Symmetric}}$ and $R_{\text {GTR}}$ represent the rate parameters of strand symmetric and general time reversible (GTR) models, respectively. Matrix indices are ordered such that $i,j\in \{1,2,3,4\}=\{A,C,G,T\}$. $\pi _{*}$ is the equilibrium distribution of the GTR model. Diagonal elements are determined by the Markov condition $\sum _{i}R_{ij}=0$

### Phylogenetic tree case

**Fig. 2:**
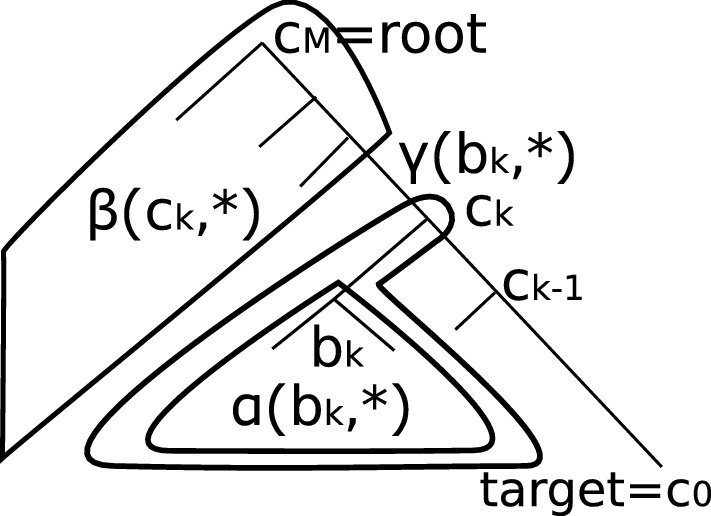
Inside and outside variables. $c_k$ denotes the concestor nodes on the target lineage. $b_k$ denotes the sibling node of $c_{k-1}$. $\alpha (b_k,*)$ represents the inside variable, while $\beta (c_k,*)$ represents the outside variable. $\gamma (b_k,*)$ represents a dynamic programming variable in Eq. 2 in the main text

To extend our algorithm to phylogenetic trees, we specify a target species that corresponds to a leaf node of a tree and consider the path from the leaf node to the root node. Each internal node along the path corresponds to the last common ancestor (*concestor*) [20] of the target species and some extant species. Let ${\mathcal {C}}={c_0,\ldots ,c_M}$ be the set of concestors with $c_M$ being the root node and $c_0$ being the leaf node of the target for convenience. Further, let $s_i$ be the fraction of path length between the leaf and $c_k$, let $s_{kl}=(s_l-s_k)$, and let ${\bar{t}}$ be the total path length from the target leaf to the root. Then, the corresponding formula of Eq. 1 is obtained by dividing the integration range into sub-intervals between neighboring concestors and inserting the probabilities $\{\gamma _k\}$ that emit partial alignment columns that are descendants of the sister branch of each concestor (see Fig. 2),

2 $$\begin{aligned} t_{\text {MRS}}&=\frac{{\bar{t}}}{{\mathcal {Z}}(Y)}\sum _b\left[ \sum _{k=1}^{M}\int _{s_{k-1}}^{s_k}e^{s_{01}{\bar{t}}R_D}\gamma _1\right.\\&\quad\left.\cdots \left[ e^{(s-s_{k-1}){\bar{t}}R_D}e^{(s_{k}-s){\bar{t}}R}\right] \gamma _k\cdots e^{s_{M-1,M}{\bar{t}}R}ds\right] _{ab}\pi _b \nonumber \\ {\mathcal {Z}}(Y)&={\mathbb {P}}(Y) \nonumber \\ \gamma _k&=\text {diag}(\gamma (b_k, 1),\ldots ,\gamma (b_k, 4)) \nonumber \\ \gamma (b_k, i)&= \sum _j\alpha (b_k,j)p(j|i, t_{b_k}) \nonumber \\ \alpha (n,i)&={\mathbb {P}}(Y({\mathcal {L}}(n))|X_{n}=i), \end{aligned}$$

where ${\mathcal {Z}}(Y)$ represents the likelihood of alignment column *Y*, $\pi$ represents the equilibrium distribution for rate matrix *R*, $Y({\mathcal {L}}')$ represents the partial alignment column for a subset of leaf nodes ${\mathcal {L}}'\in {\mathcal {L}}$ ( ${\mathcal {L}}$: the set of all leaf nodes), $a=Y(c_0)$, $b_k$ represents the sibling node of $c_{k-1}$ with parent node $c_k$, $t_n$ represents the edge length between node *n* and its parent node, ${\mathcal {L}}(n)$ the descendant leaves of node *n*, and $X_{n}$ represents the random variable that represents the nucleotide type at node *n*. The inside variable $\alpha (n,i)={\mathbb {P}}(Y({\mathcal {L}}(n))|X_{n}=i)$ represents the probability of emitting partial alignment column $Y({\mathcal {L}}(n))$ given the state at node *n* is fixed to *i*. See Fig. 2 for the relations between tree nodes and dynamic programming variables. Because the range of integration is localized only in the *k*-th edge in the above equation, we can compute $t_{\text {MRS}}$ using a dynamic programming algorithm (Algorithm 1). In 
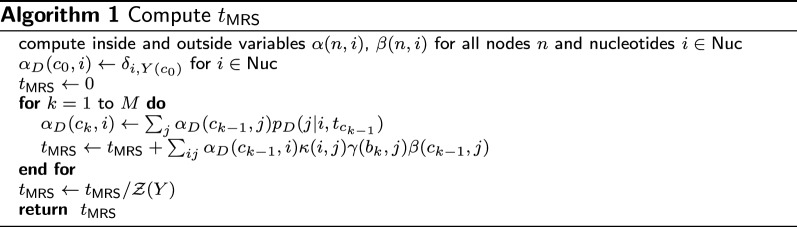
 Algorithm 1, $p_D(j|i,t_{c_{k-1}})=\left[ \exp (tR_D)\right] _{ji}$ represents the probability of transition $j\leftarrow i$ after time *t* without any substitution. $\kappa (i,j)$ is defined by

$$\begin{aligned} \kappa (i,j)={\bar{t}}\sum _l U_{il}{U^{-1}}_{lj}{\mathcal {K}}(s_{k-1,k}{\bar{t}}R_{Dii}, s_{k-1,k}{\bar{t}}\lambda _l). \end{aligned}$$

$\beta (n,i)={\mathbb {P}}(Y({\mathcal {L}}\backslash {\mathcal {L}}(n)),X_{\text {Pa}(n)}=i)$ is called an outside variable and represents the probability of emitting alignment nucleotides other than the descendants ${\mathcal {L}}(n)$ of node *n* with a constraint that the state of the parent node $\text {Pa}(n)$ is fixed to *i*. The inside and outside variables are computed by using the inside and outside algorithms [4, 19] resembling the use of forward-backward algorithms in linear hidden Markov models. $\alpha _D(c_k,i)$ is the probability that emits the partial alignment column $Y({\mathcal {L}}(n))$ with no substitution along the target lineage up to concestor node $c_k$, given the state of node *n* is fixed to *i*.

Similar algorithms can be derived for the standard deviation $\sigma$ and the probability of no mutation *q* as described in Additional file 1.

### Alignment gaps and ambiguous characters

We treat gap and ambiguous nucleotide characters of non-target leaves as missing characters; we sum the probabilities of all possible nucleotide patterns in computation. Then, the probability condition indicates that the estimated values are the same as those computed from the reduced phylogenetic tree and alignment columns after removal of gaps and ambiguous characters and the corresponding edges in the tree. This increases the standard deviation $\sigma$ of estimates $t_{\text {MRS}}$. On the other hand, we do not consider the sites if the character of the target is a gap or an ambiguous character.

### Software availability

We implemented our algorithms in the C++ language. The resulting software (‘TMRS’) is available at our website [21].

## Dataset and data processing

We downloaded the MAF-formatted Multiz100way multiple alignment files from the UCSC genome browser site, which consists of multiple genome alignments of 100 vertebrate species, including the human genome version hg38. We also downloaded the phylogenetic tree data from the PhyloP track, whose edge lengths are trained using fourfold degenerate (4d) sites of RefSeq genes under the general time reversible model.

We used the topology of the PhyloP phylogenetic tree as it is, and trained only the edge lengths of the tree as well as the rate parameters of the strand symmetric model. For this, we collected alignment columns at human 4d sites based on gene annotations of the RefGene track from the UCSC site, following Siepel et al. [8] and Pollard et al. [9]. The reason for using 4d sites is the higher quality of alignments and higher coverage of distant species in the alignments [8, 9], though they may be subject to various evolutionary constraints. In order to investigate the uncertainty of trained parameters, we randomly sampled 100 sets of 4d sites from about three million 4d sites in the human genome such that each has a given number of sites, ranging from 1 to $10^5$. We generated an alignment of concatenated genomic alignment columns, and trained parameters based on the maximum likelihood method [22], using the LBFGS-B gradient descent package [17].

For studying differences in $t_{\text {MRS}}$ distributions among genes, we sampled 100,000 alignment columns from intergenic, CDS, 3′UTR, and 5′UTR sequences based on ‘Gencode v24 Basic’ track gene models from the UCSC site [3].

Anderson et al. [23] identified genomic elements called transcribed enhancers in human and other genomes, where short RNAs are produced by bidirectional transcription as a result of chromatin openings. From the FANTOM5 enhancer atlas site [24], we downloaded the coordinates of transcribed enhancers and the list of tissue and cell specific enhancers where bidirectional transcription occurs in a tissue and/or cell-specific manner.

## Results and discussions

### Parameter optimization and performance tests

**Fig. 3:**
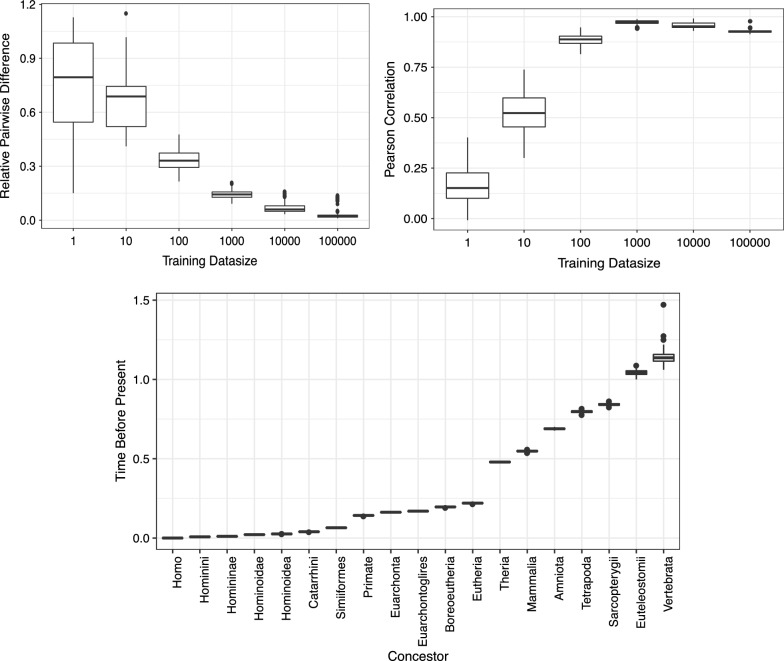
Convergence of optimized parameters. The upper left panel shows the distributions of the pairwise relative differences of inferred parameters. The *x*-axis represents the number of alignment columns used to train the parameters. The upper right panel represents the distribution of the correlation coefficients of tree edge lengths between the PhyloP model of the UCSC genome browser site and the inferred parameters. The *x*-axis is the same as that shown in the upper left panel. The bottom panel represents the distributions of inferred time to each concestor from the present. The unit is the number of substitutions per site. Each parameter set is trained using 100,000 alignment columns sampled from 4d sites

We trained rate matrix $\{R_{ij}\}$ and tree edge lengths $\{t_k\}$ from genomic multiple alignment columns sampled from 4d sites. We trained 100 sets of parameters with random initial points from 100 sets of random-sampled alignment columns. Figure 3 (top left) shows the distributions of pairwise relative differences of trained parameters $\theta = \left(\{R_{ij}\},\{t_k\} \right)$ for each number of alignment columns. Here, the relative difference between two parameters $\theta _1$ and $\theta _2$ is defined by $|\theta _1 - \theta _2| / \text{max} (|\theta _1|, |\theta _2|)$ with |*v*| being the Euclidean norm. It shows the trained parameter converges very well as increasing the number of alignment columns. Figure 3 (top right) shows the Pearson correlation coefficient with the tree edge lengths provided in the PhyloP track of the UCSC genome browser, which was computed using the general time reversible model [9]. It shows concordant tree edge lengths (correlation coefficient $> 0.9$) are learned despite the differences in rate matrix models. Figure 3 (bottom) shows the distributions of the tree path lengths from the leaf node of humans to its concestor nodes using parameters trained with 100,000 alignment columns. As the variance among training sets is very small, we use their mean values as the times to concestors before the present and do not consider the widths of distributions. Table 2 shows the mean rate matrix and equilibrium distribution. The average transition-transversion rate ratio is about 2.7 in this model (see Section 6 in Additional file 1 for the computation). In the following results, we use 100 sets of parameters that are trained from 100,000 alignment columns and take averages of $t_{\text {MRS}}$, $\sigma$, and *q* computed for each parameter set.

**Table 2:** Trained rate matrix and equilibrium distribution

Substitution type	Parameter	Rate
$\text {A}\leftarrow \text {C}$, $\text {T}\leftarrow \text {G}$	$\alpha$	0.16
$\text {A}\leftarrow \text {G}$, $\text {T}\leftarrow \text {C}$	$\beta$	0.57
$\text {A}\leftarrow \text {T}$, $\text {T}\leftarrow \text {A}$	$\gamma$	0.20
$\text {C}\leftarrow \text {G}$, $\text {G}\leftarrow \text {C}$	$\delta$	0.24
$\text {C}\leftarrow \text {T}$, $\text {G}\leftarrow \text {A}$	$\epsilon$	0.59
$\text {G}\leftarrow \text {T}$, $\text {C}\leftarrow \text {A}$	$\eta$	0.25

Nucleotide	Equilibrium frequency $\pi$	
A, T	0.23	
C, G	0.27	

The elements of rate matrix $R_{\text {Symmetric}}$ and its equilibrium frequency $\pi$ are shown. Parameter variables correspond to matrix $R_{\text {Symmetric}}$ in Table 1. We averaged the parameters optimized using 100,000 sampled alignment columns in the 4d sites. Due to the symmetry of rate matrix, complementary nucleotides have the same equilibrium frequency

**Fig. 4:**
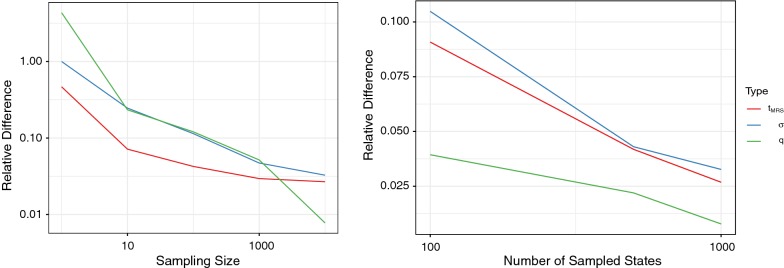
Numerical tests of our algorithms. The statistics $t_{\text {MRS}}$, $\sigma$, and *q* computed by exact algorithms were compared with those estimated using sampled histories of nucleotide substitutions. The *y*-axes represent the relative difference between the values from the exact algorithms and those obtained by approximate sampling algorithms. The *x*-axes show the dependency on the number of sampled histories and the number of discrete points in the phylogenetic tree from which the states were sampled

In Fig. 4, we compared ($t_{\text {MRS}}$, $\sigma$, *q*) computed by our algorithms with the corresponding values obtained from posterior sampling of mutation histories along the phylogenetic tree in order to numerically check the correctness of our algorithms. It shows the relative errors between two values monotonically decrease as the sample size and the fineness of discretization increases.

Table 3 shows the runtimes of our C++ implementation. We used a single ES-2670 v3 2.3 GHz core as the computational platform. As the $t_{\text {MRS}}$, $\sigma$, and *q* values of each alignment columns are independently computable, our algorithms can deal with the entire human genome with reasonable time using a compute cluster.

**Table 3:** Runtime of our implementation

Computation	Datasize	Runtime
Train, gradient (1 iteration)	100 K columns	4.6 min
Train, total (300 iterations)	100 K columns	23 h
$t_{\text {MRS}}$, $\sigma$, *q*	1 column	$7.3\times 10^{-4}$ s
$t_{\text {MRS}}$, $\sigma$, *q*	1 G columns	204 h

We show runtimes of our implementation. We used 100 species vertebrate multiple alignments for the measurements. For training data, we used a sampled alignment with 100 K columns from 4d sites. As for the computation of $t_{\text {MRS}}$, $\sigma$, and *q*, we used the sampled alignments from 3$'$UTR sequences which have 2,034,681 total alignment columns, and scaled the runtime for each Datasize

### Comparison with other statistical measures

To show the significance of our algorithms, we compared the accuracy with two possible methods of estimating $t_{\text {MRS}}$ and *q*. The first method (termed ‘reconstruction’) uses the ancestral reconstruction. In this method, we first set the nucleotide state of each concestor node $c_k$ to the base $a_{c_k}$ with the maximal posterior probability:

$$\begin{aligned} a_{c_k}&= \text {argmax}_{i}{\mathbb {P}}\left( X_{c_k}=i|Y\right) =\frac{1}{{\mathcal {Z}}(Y)}\sum _j\alpha (c_k, i)p(i|j,t_{c_k})\beta (\text {Pa}(c_k),j) \end{aligned}$$

Then, we return the middle point of the edge between nodes $c_{k-1}$ and $c_k$ as $t_{\text {MRS}}$ where $c_k$ is the most recent concestor whose reconstructed nucleotides differ from that of the target species $a_{c_k}\ne Y(c_0)$. We set $q=1$ if there is no such $c_k$ and we set $q=0$ otherwise. The second method (termed ‘alignment’) to infer $t_{\text {MRS}}$ only considers nucleotides of extant species: we return the middle point of the edge between nodes $c_{k-1}$ and $c_k$ as $t_{\text {MRS}}$ where $c_k$ is the most recent concestor such that partial alignment column $Y({\mathcal {L}}(c_k)$, which are descendants of $c_k$, contain different nucleotide from the target nucleotides $\exists a\in Y({\mathcal {L}}(c_k)), a\ne Y(c_0)$. Similarly to the ‘reconstruction’ method, We set $q=1$ if there is no such $c_k$ and we set $q=0$ otherwise.

To compare the accuracy of our algorithm with these approximate algorithms, we simulated evolutionary history and alignment column of base mutation using forward simulation using the phylogenetic model of the previous section. We masked nucleotide positions where there are gap or ambiguous characters in sampled multiz100way alignments in order to imitate the gap patterns of real alignments. Details of the simulation algorithm is described in Section 6 of Additional file 1. As a result, we obtained 100,000 alignment columns of 100 species with ‘true’ annotation of $t_{\text {MRS}}$ and $q\in \{0,1\}$.

**Fig. 5:**
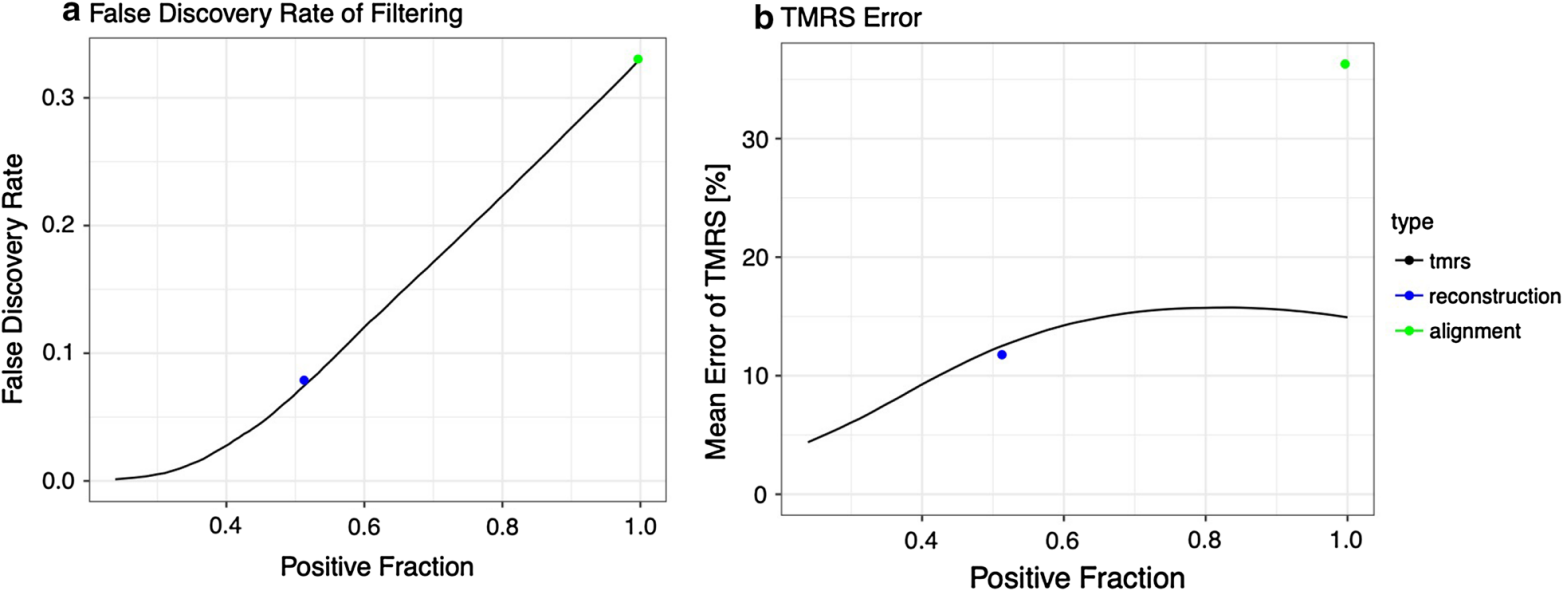
Effect of filtering. We investigated the effect of filtering by *q* threshold on the accuracy of $t_{\text {MRS}}$ estimates using simulation dataset. The *x*-axis represents the fraction of alignment columns remained by filtering with varying threshold.** a** Fraction of alignment columns that have no mutation throughout the target lineage in the positive set.** b** Mean % error of $t_{\text {MRS}}$ values in the dataset after filtering. The blue and green points represent the approximate $t_{\text {MRS}}$ and *q* computed from the reconstruction of ancestral states, and the closest extant species whose base is different from that of the target species, respectively

Figure 5a shows accuracies of predicting the absence of mutation along the target lineage. The *x*-axis is the fraction of positives in the dataset which was controlled by varying threshold of *q*. Since the ’reconstruction’ and ’alignment’ methods assign only binary *q* values, only a single point is plotted for each. The *y*-axis represents the ratio of false positives in all the positive predictions (i.e. False Discovery Rate, FDR). It shows that FDR monotonically decreases with decreasing *q* threshold, indicating the correctness of our algorithm for *q*. It also indicates that the accuracies of absence call of reconstruction and alignment methods are similar to that of our algorithm with positive fraction 0.5 and 1.0, respectively. Figure 5b shows the mean errors of predicted $t_{\text {MRS}}$ relative to the total length of target lineage for each positive fraction. The error mostly decreases with stricter thresholds for our method, while reconstruction and alignment methods show more than 10% errors on average. Table 4 shows numerical values of FDR and mean error for several *q* threshold. Since the mean error of $t_{\text {MRS}}$ is less than 5% of the total length of target lineage, we will use threshold $q=0.01$ in the analyses in the following sections.

**Table 4:** Effects of filtering by probability *q* of no mutation

*q* threshold	Positive fraction	FDR for no mutation	% error of $t_{\text {MRS}}$
0.01	0.24	0.0013	4.4
0.1	0.36	0.015	7.9
0.5	0.68	0.16	15
1.0	1	0.33	15

We computed a few statistical measures for the simulation dataset obtained by forward sampling of base mutation history. The first column represents the threshold values $q_{\text {threshold}}$. ‘positive fraction’ represents the fraction of alignment columns with $q < q_{\text {threshold}}$. ‘FDR for no mutation’ represents the fraction of the alignment columns that have no mutation along the target lineage but satisfy $q<q_{\text {threshold}}$. ‘% error of $t_{\text {MRS}}$’ represents the mean % error of estimated $t_{\text {MRS}}$ relative to the total edge length of the target lineage

Table 5 shows the comparison of $t_{\text {MRS}}$ and other statistical measures computed from genomic alignments. We used the same alignment columns in the previous paragraph but with filtering with threshold $q < 1$ for true *q* values. For this dataset, we computed Spearman’s correlation coefficients with true $t_{\text {MRS}}$ and other indicators: $t_{\text {MRS}}(q<0.01,0.1,1)$ represents our algorithms with a few filtering criteria of *q*. ‘reconstruction’ and ‘alignment’ are the approximate methods described above with filtering based on *q* values computed by their respective method. ‘entropy’ represents the information entropy of base frequency of alignment column. ‘pairwise’ represents the ratio of the number of identical bases in $n(n-1)/2$ possible base pairs of *n* bases in the alignment column. ‘phastcons’ represents the posterior probability of conserved region computed by PhastCons [8]. ‘phylop’ represents the negative p-value of conservation computed by PhyloP [9]. ‘gerp’ represents the estimated number of ‘rejected mutations’ compute by Gerp++ [10]. The table shows small correlation of conservation measures (entropy, pairwise, phastcons, phylop, gerp) with $t_{\text {MRS}}$ and very high correlation of estimated $t_{\text {MRS}}$ with strict filtering criterion $q<0.01$. It shows our algorithms can accurately extract distinct evolutionary information which is difficult to extract with previous conservation measures.

**Table 5:** Correlation with other conservation measures

Significance measure	Spearman’s correlation with true $t_{\text {MRS}}$
$t_{\text {MRS}}(q<0.01)$	0.965
$t_{\text {MRS}}(q<0.1)$	0.938
$t_{\text {MRS}}(q<1)$	0.858
Reconstruction ($q<1$)	0.905
Alignment ($q<1$)	0.338
Entropy	0.301
Pairwise	0.344
Phastcons	0.112
Phylop	0.108
Gerp	0.129

We show Spearman’s correlation coefficient with the true $t_{\text {MRS}}$ obtained from simulation and several measures for nucleotide conservation. The first three columns represent $t_{\text {MRS}}$ computed by our exact algorithm with filtering by *q* values. ‘reconstruction’ represents approximate $t_{\text {MRS}}$ values estimated from reconstruction of ancestral states. ‘alignment’ represents approximate $t_{\text {MRS}}$ values estimated from closest extant species which has different nucleotide base from the nucleotide of the target species. ‘entropy’ represents the negative information entropy of the base frequency of alignment column. ‘pairwise’ represents the pairwise alignment similarity of alignment column. ‘phastcons’ represents the posterior probability of conservation at the alignment column. ’phylop’ represents the p-values of negative selection. ‘gerp’ represents the ‘rejected substitution’ values

### Genomic distribution of $t_{\text {MRS}}$

**Fig. 6:**
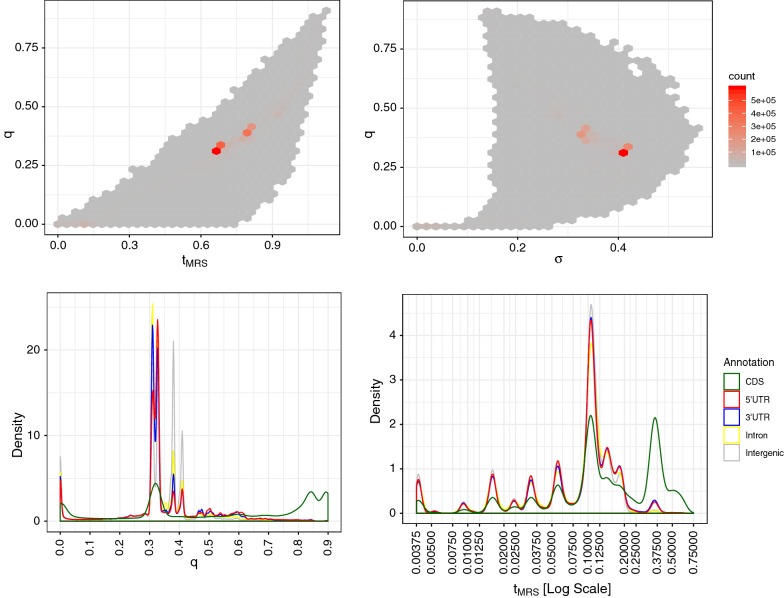
Distributions of $t_{\text {MRS}}, \sigma$ and* q* in the human genome. The top panels show the sampling distribution of statistics $t_{\text {MRS}}$-*q* (left) and $\sigma$-*q* (right) in the human genome. In these panels, a total of 2,063,207 alignment columns were sampled from the human genome excluding repeat regions. The bottom panels show the densities of *q* (left) and $t_{\text {MRS}}$ (with $q<0.01$) (right) for several types of genomic region: CDS, 5$'$UTR, 3$'$UTR, Intron, and Intergenic

We computed the time to the most recent substitution $t_{\text {MRS}}$, its standard deviation $\sigma$, and the probability *q* that there is no substitution for alignment columns uniformly sampled from the human genome. The scatter plot of $t_{\text {MRS}}$ and *q* values (Fig. 6 (top left)) shows the probability of no substitution *q* tends to increase with increasing $t_{\text {MRS}}$. However, the distribution is broad depending on the nucleotide patterns of alignment columns, and a non-zero fraction of sites have deep ancestral substitutions (i.e., large $t_{\text {MRS}}$ and small *q*) within the Homo–Vertebrate lineage. The scatter plot of $\sigma$ and *q* (Fig. 6 (right)) shows that the probability of no substitution *q* is very small if $\sigma < 0.1$. The high peaks (the red regions) of these two figures show that a large number of alignment columns have $t_{\text {MRS}}\sim 0.7$, $\sigma \sim 0.4$, and $q\sim 0.3$. For these sites, it is difficult to determine if there are substitutions within the interval of the Homo–Vertebrate lineage.

**Table 6:** Evolutionary time of reduced concestors

Concestor	Time	Sibling	Descendants
Homo	0	–	Human
Hominoidea	0.026	Hylobatidae	Gibbon
Euarchontoglires	0.17	Glires	Mouse, rabbit
Eutheria	0.22	Atlantogenata	Elephant, armadillo
Mammalia	0.55	Prototheria	Platypus
Amniota	0.69	Sauropsida	Bird, reptile
Tetrapoda	0.80	Amphibia	Frog
Vertebrata	1.1	Cyclostomata	Lamprey

**Fig. 7:**
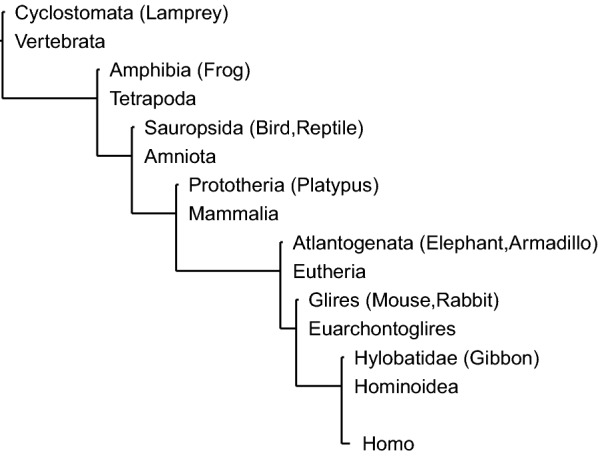
Topological relationship of reduced concestors. We show the topology of simplified phylogenetic tree of 100 vertebrate species used in the analyses of concestor intervals. See Table 6 for the numerical values of evolutionary time

We used the following set of reduced concestors in the analyses of concestor intervals. Each concestor was named based on the corresponding taxonomic class of descendants. ‘Time’ represents phylogenetic time from the present in units of substitutions per site. ‘Sibling’ represents the sibling clade that departed from the human lineage at each concestor. ‘Descendants’ represent some examples of extant species in the sibling clade. Topological relationships are shown in Fig. 7

Figure 6 (bottom left) shows the density of *q* for each annotated genomic region. Compared to Intergenic, Intron, 3′UTR, and 5′UTR, CDS regions have a large fraction of sites with a high probability of no mutation, indicating many ancestral nucleotides that were fixed before the appearance of the vertebrate concestor. Since computed $t_{\text {MRS}}$ values have less meaning if *q* is large, we filtered out sites with $q>0.01$ and plotted the distributions of $t_{\text {MRS}}$ values for the remaining sites (Fig. 6 (bottom right)). There are several peaks because some sites are guaranteed to experience the last substitution between specific interval of concestors. All regions have the highest peak around $t_{\text {MRS}}\sim 0.1$, which is between the Simiiformes and Primate concestors. CDS regions have a large peak around $t_{\text {MRS}}\sim 0.36$, which corresponds to between the Eutheria and Theria concestors.

### Concestor interval of the last substitution event

**Fig. 8:**
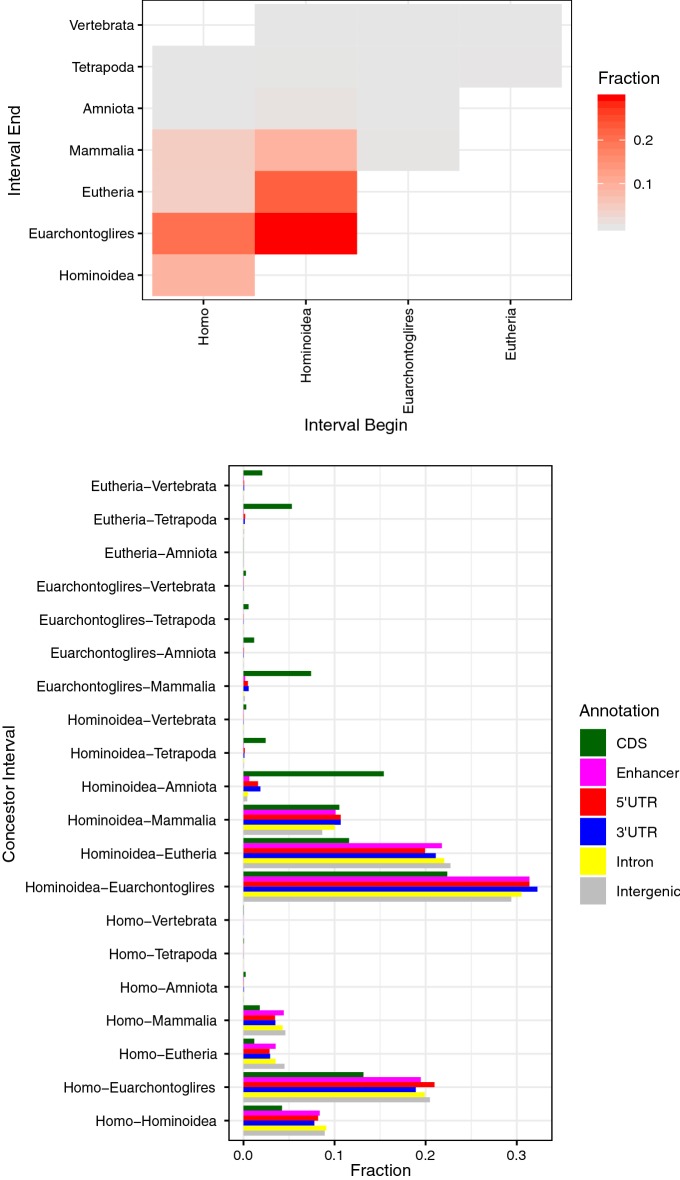
Frequency of concestor intervals. The two panels show the frequencies of genomic sites categorized by the concestor intervals where their most recent substitutions occurred. The left panel shows the genomic distribution. The axes represent late (*x*-axis) and early (*y*-axis) ends of intervals. The right panel shows distributions for several types of genomic region: CDS, 5′UTR, 3′UTR, Intron, Intergenic, and Transcribed Enhancer. Only intervals with non-zero counts are shown in this panel

We are generally interested in the substitutions that are associated with the evolution of unique features in the species that inherited them. In this respect, we want to know in which interval between two speciation events (i.e., between two concestor nodes) each $t_{\text {MRS}}$ is located. In order to simplify the presentation, we reduced the concestor nodes from the full 19 concestors of the PhyloP tree to eight as shown in Table 6 and Fig. 7 in the following analyses of concestor intervals. Since the estimated $t_{\text {MRS}}$ values can have a large standard deviation $\sigma$, we consider intervals between all pairs of concestor nodes: Homo–Hominoidea, Homo–Mammalia, Mammalia–Vertebrata, etc. Then, we assign a concestor interval *I* to a site if $q<0.01$ and if *I* is the smallest interval that contains a confidence interval $[t_{\text {MRS}}- 2\sigma , t_{\text {MRS}}+ 2\sigma ]$. Only about 4% of sites were assigned to any concestor interval by this method. Figure 8 (top) shows the frequency distribution of genomic sites that are assigned to some concestor interval, which shows that many sites are assigned to concestor intervals Hominoidea–Euarchontoglires, Hominoidea–Eutheria, or Homo–Euarchontoglires. Figure 8 (bottom) shows the same frequency distributions for each category of annotated genomic regions. The distributions, except that of CDS, are similar to each other. On the other hand, CDS regions have many deep ancestral intervals.

### Tissue-concestor interval correlations for transcribed enhancers

**Fig. 9:**
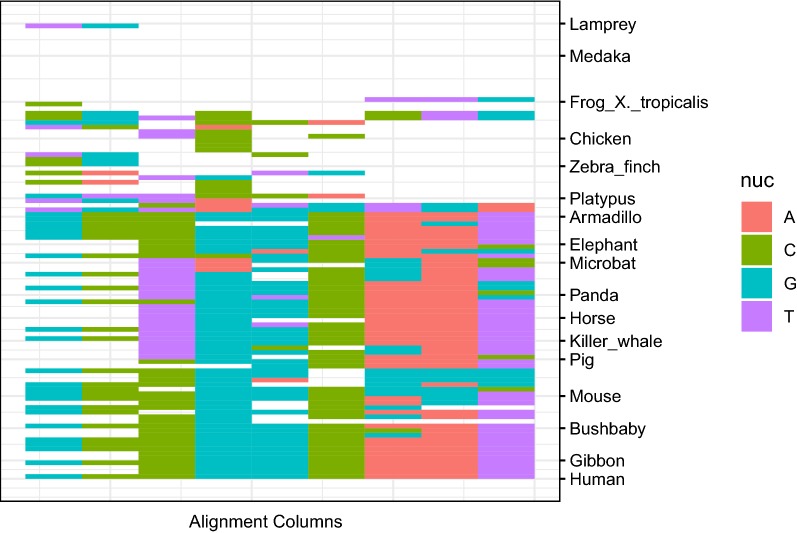
Examples of alignment columns. The figure shows the examples of alignment columns that include the concestor interval Homo–Vertebrata in the transcribed enhancer regions and show brain-specific RNA transcription. The *y*-axis represents nine example alignment columns and *x*-axis represents nucleotides of each column, in which gaps, ambiguous nucleotides, and unaligned regions are shown as blank. The species are aligned such that it conforms phylogenetic trees and sorted such that species more evolutionarily distant from humans are placed on the right

Andersson et al. [23] identified genomic elements called transcribed enhancers in the human genome and other genomes where short RNAs are produced by bidirectional transcription as a result of chromatin opening. They showed transcribed enhancers often overlap with protein-binding marks such as ChIP-seq peaks or protein-binding motifs. They are also enriched in disease-associated single nucleotide polymorphisms (SNPs). Many transcribed enhancers are tissue-specific in that bidirectional transcription of short RNAs occurs frequently in specific tissues. They showed the expressions of a number of genes are well explained by those of a few transcribed enhancers upstream of the genes. Thus, we can see tissue specific enhancer activities for these transcribed enhancers. In the FANTOM5 enhancer atlas site [24], tissue-specific enhancers are annotated by using the UBERON tissue anatomy ontology and Cell Ontology [25, 26]. For example, 41 diverse tissues were assigned to 10-1335 differentially-expressed enhancers (see Additional file 1: Table S2) [24]. Using these data, we studied the tissue and concestor interval of the last substitution event as an example of screening evolutionarily important events that affected life designs of extant organisms. We computed $(t_{\text {MRS}}, \sigma , q)$ for each site of the transcribed enhancer regions, filtered out the sites with $q>0.01$, and associated concestor intervals as described above. For each concestor interval, we list the enhancers that contain sites associated with the interval. We used the hypergeometric test to determine if the sites corresponding to a specific concestor interval are significantly enriched for the enhancers transcribed in a specific tissue type. Table 7 shows tissues that have the top five most significant *p*-values for some concestor interval (more details are discussed in Section 7 of Additional file 1). We find that the brain and Homo–Vertebrata interval association has the most significant *p*-value and Homo–Vertebrata sites are enriched threefold in brain-associated enhancers relative to expectations. The second tissue was meninx, which is also associated with the nervous system (Table 7). Figure 9 shows a few sampled alignment columns in a brain-specific enhancer, which are assigned to the Homo–Vertebrata interval. Alignment columns that have three or more nucleotides suggest there are some substitutions along the Homo–Vertebrata lineage, but the patterns of nucleotide types and the number of gaps makes it difficult to determine at what time point the substitution occurs. Thus, the assigned intervals are the most ambiguous for these alignment columns.

**Table 7:** Tissue-concestor interval correlation for transcribed enhancers

Tissue	Interval	Z-score	$-\log _{10}(\text {p-value})$	Enrichment	Observed
Brain	Homo–Vertebrata	14.2	32.0	2.97	140
	Hominoidea–Tetrapoda	13.3	29.2	2.69	148
	Hominoidea–Vertebrata	11.5	22.1	2.17	115
Meninx	Hominoidea–Tetrapoda	8.58	11.7	3.84	32
	Hominoidea–Vertebrata	6.68	7.72	3.52	23
	Hominoidea–Amniota	6.06	6.96	3.01	25
Eye	Hominoidea–Vertebrata	8.43	11.5	3.50	37
	Eutheria–Tetrapoda	8.42	7.44	9.69	9
	Eutheria–Vertebrata	8.06	8.98	5.13	19

The top three tissues that have transcribed enhancers with sites corresponding to specific concestor intervals are shown. Within each tissue, the top three concestor intervals are shown. The sorting order is based on *Z*-scores that are based on the hypergeometric test and indicate the significance of enrichment of specific concestor intervals in tissue-specific enhancers. ‘$-\log _{10}(\text {p-value})$’ is the minus log10 *p*-value of the test computed using the phyper() function in the R programming language. ‘Enrichment’ is the fold enrichment within the concestor interval relative to the expected occurrence by random sampling. ‘Observed’ is the number of transcribed enhancers that have both attributes of the Tissue and Interval columns. see Section 8 in Additional file 1 about the use of *Z*-scores for ranking tissues

### Tissue-concestor interval correlations for genes

We studied the correlation between the tissue-specificity and concestor intervals for genes in a similar manner as for transcribed enhancers. See Section 9 in Additional file 1 for detailed description of the method. Table 8 shows the top three tissues that have genes with sites corresponding to specific concestor intervals are shown. Within each tissue, the top three concestor intervals are shown. As compared to the corresponding Table 7 for transcribed enhancers, deeply ancestral intervals appear in the table, indicating the high level of conservation of exonic sequences. On the other hand, fold enrichment of concestor intervals are smaller than in transcribed enhancers which make it more difficult to infer the impact of the most recent mutations on the life design of extant species than in the case of transcribed enhancers.

**Table 8:** Tissue-concestor interval correlation for genes

Tissue	Interval	Z-score	$-\log _{10}(\text {p-value})$	Enrichment	Observed
Muscle	Eutheria–Tetrapoda	6.44	11.3	1.26	250
	Eutheria–Amniota	6.23	11.9	1.18	287
	Eutheria–Mammalia	5.22	10.8	1.09	307
Artery aorta	Eutheria–Tetrapoda	5.73	9.76	1.36	122
	Eutheria–Amniota	4.22	6.20	1.18	131
	Euarchontoglires–Eutheria	4.16	5.11	1.33	99
Pineal gland	Euarchontoglires–Eutheria	5.50	8.05	1.28	217
	Eutheria–Amniota	5.45	9.04	1.15	290
	Eutheria–Tetrapoda	5.21	7.60	1.21	247

The top three tissues that have genes with sites corresponding to specific concestor intervals are shown. Within each tissue, the top three concestor intervals are shown. The sorting order is based on *Z*-scores that are based on the hypergeometric test and indicate the significance of enrichment of specific concestor intervals in tissue-specific genes. ‘$-\log _{10}(\text {p-value})$’ is the minus log10 *p*-value of the test computed using the phyper() function in the R programming language. ‘Enrichment’ is the fold enrichment within the concestor interval relative to the expected occurrence by random sampling. ‘Observed’ is the number of Entrez genes that have both attributes of the tissue and interval columns

## Conclusions

We have developed algorithms to infer the time $t_{\text {MRS}}$ to most recent substitution in the lineage from a given target species to the root of a phylogenetic tree. In order to filter out highly conserved sites and ambiguous sites where the confidence of estimated $t_{\text {MRS}}$ is low, we also compute the probability *q* of no mutation and the standard deviation $\sigma$ of $t_{\text {MRS}}$. We computed these variables efficiently using dynamic programming algorithms on the phylogenetic tree such that the algorithms can be applied to multiple genomic alignments with 100 species. We have empirically checked the correctness of our algorithms by posterior sampling of mutation histories on the tree. Our algorithms are exact under the assumptions of the model: genome evolution follows a site-independent continuous-time Markov process along the phylogenetic tree. Our results also depend on the quality of Multiz alignment, which was debated previously [27]. Although alignment errors can be less influential if the corresponding leaf nodes are far from the target lineage, the incomplete coverage of sequenced genomes directly affects the number of sites whose $t_{\text {MRS}}$ can be determined with confidence. We expect that the number of sites with confident $t_{\text {MRS}}$ value will increase as the coverage of genome sequences improve in the future.

We have applied our tool to 100-species multiple genome alignments with human target and obtained a frequency spectrum of concestor intervals that categorized the time points at which the last substitutions occurred. Furthermore, we studied the correlation between the frequency of concestor intervals and the tissue-specificity of transcribed enhancers and found that brain-specific transcribed enhancers are highly enriched among the sites with mutations in the human lineage. It may be very interesting to combine our method with genome editing experiments to see if nucleotide changes at the screened sites affect tissue functions.

## Supplementary information

**Additional file 1.** Detailed description of TMRS algorithms.
