# Supplementary material for: TMRS: an algorithm for computing the time to the most recent substitution event from a multiple alignment column
Source: Algorithms Mol Biol. 2019 Nov 18;14:23. doi: 10.1186/s13015-019-0158-3 (PMC6859643; doi:10.1186/s13015-019-0158-3)
Supplement: Supplementary file 1 — Additional file 1. Detailed description of TMRS algorithms. [file 13015_2019_158_MOESM1_ESM.pdf]

Additional file 1 for TMRS: an algorithm for computing the time to the most recent substitution event from a multiple alignment column

Hisanori Kiryu, Yuto Ichikawa, Yasuhiro Kojima

November 14, 2019

1 Derivation of the Algorithms for σ and q

In order to compute the standard deviation of T_{MRS} in the main text, we need the expression for T^2_{MRS}, which is given by

$$\begin{aligned} T^2_{\mathrm{MRS}} &= \left(\frac{t}{N}\right)^2 \sum_{l=1}^{N}\sum_{u=1}^{N} \mathbb{I}(X_N = \cdots = X_{N-l})\mathbb{I}(X_N = \cdots = X_{N-u}) \\ &= \left(\frac{t}{N}\right)^2 \sum_{l=1}^{N} (2l-1)\mathbb{I}(X_N = \cdots = X_{N-l}), \end{aligned}$$

where we use $\mathbb{I}(X_N = \cdots = X_k)\mathbb{I}(X_N = \cdots = X_l) = \mathbb{I}(X_N = \cdots = X_{\min(k,l)})$ and collect terms with the same indicator function. The expected value of T^2_{MRS} is given by

$$\mathbb{E}\left(T^2_{\mathrm{MRS}}|a,b,t\right) = \left(\frac{t}{N}\right)^2 \sum_{l=1}^{N-1} (2l-1) \left[Q_D^l Q^{N-l}\right]_{ab} / \left[Q^N\right]_{ab}.$$

To take the continuum limit $N \to \infty$, we make the following substitution

$$\begin{aligned} \sum_{l=1}^{N} \frac{1}{N} f\left(\frac{l}{N}\right) &\to \int_0^1 f(s)ds \\ Q_D^l &\to \exp(stR_D), (s = l/N) \\ Q^{N-l} &\to \exp((1-s)tR) \\ \frac{2l-1}{N} &\to 2s. \end{aligned}$$

Then we have the continuous version of $\mathbb{E}\left(T^2_{\mathrm{MRS}}|a,b,t\right)$,

$$\begin{aligned}
\mathbb{E}\left(T^2_{\mathrm{MRS}}|a,b,t\right) &= \frac{2t^2}{\mathcal{Z}}\left[\int_0^1 s e^{stR_D} e^{(1-s)tR} ds\right]_{ab} \\
&= \frac{2t^2}{\mathcal{Z}}\sum_{i=1}^{4} U_{ai}U^{-1}{}_{ib}\mathcal{K}'(tR_{Daa}, t\lambda_i) \\
\mathcal{Z} &= \left[e^{tR}\right]_{ab} = \sum_{i=1}^{4} U_{ai}e^{t\lambda_i}U^{-1}{}_{ib} \\
\mathcal{K}'(x,y) &= \int_0^1 s e^{sx}e^{(1-s)y}ds = \frac{\partial}{\partial x}\int_0^1 e^{sx}e^{(1-s)y}ds = \frac{\partial}{\partial x}\mathcal{K}(x,y).
\end{aligned}$$

The standard deviation $\sigma(a,b,t)$ is given by

$$\sigma(a,b,t) = \sqrt{\mathbb{E}\left(T^2_{\mathrm{MRS}}|a,b,t\right) - t^2_{\mathrm{MRS}}(a,b,t)}.$$

The probability that there has been no substitution is given by

$$\mathbb{P}(X_N = \cdots = X_0|a,b,t) = \frac{\left[Q_D^N\right]_{ab}}{\left[Q^N\right]_{ab}}.$$

It has a simple $N \to \infty$ limit,

$$q(a,b,t) = \frac{\left[e^{tR_D}\right]_{ab}}{\mathcal{Z}} = \frac{e^{tR_{Daa}}\delta_{ab}}{\mathcal{Z}}$$

2 Phylogenetic Tree Case

As in the case of t_{MRS}, we divide the integration for $\mathbb{E}\left(T^2_{\mathrm{MRS}}\right)$ over the target lineage into intervals separated by concestor nodes and insert the probabilities γ_k that correspond to the probabilities of emitting a partial alignment columns that are the descendants of the sibling node of the target lineage.

$$\mathbb{E}\left(T^2_{\mathrm{MRS}}\right) =$$
$$\frac{2\bar{t}^2}{\mathcal{Z}(Y)}\sum_b\left[\sum_{k=1}^{M}\int_{s_{k-1}}^{s_k}\left[\begin{array}{c}\{s_{01}+\cdots+s_{k-2,k-1}+(s-s_{k-1})\}\times \\ e^{s_{01}\bar{t}R_D}\gamma_1\cdots\left[e^{(s-s_{k-1})\bar{t}R_D}e^{(s_k-s)\bar{t}R}\right]\gamma_k\cdots e^{s_{M-1,M}\bar{t}R}\end{array}\right]ds\right]_{ab}\pi_b.$$

As in the main text,

$$\begin{aligned}
\mathcal{Z}(Y) &= \mathbb{P}(Y) \\
\gamma_k &= \mathrm{diag}(\gamma(b_k,1),\ldots,\gamma(b_k,4)) \\
\gamma(b_k,i) &= \sum_j \alpha(b_k,j)p(j|i,t_{b_k}) \\
\alpha(n,i) &= \mathbb{P}(Y(\mathcal{D}(n))|X_n = i)
\end{aligned}$$

In the above formulas, $\bar{t}$ represents the total edge length from the target leaf to the root node, Y represents an alignment column, $\mathcal{Z}(Y)$ represents the likelihood of Y given the parameters, s_k represents the fraction of edge length from the leaf to concestor c_k relative to the total length of lineage $\bar{t}$, $s_{ij} = (s_j - s_i)$, R_D represents the diagonal part of rate matrix R, b_k represents the sibling node of concestor c_{k-1} with the common parent c_k, π represents the equilibrium probability distribution of rate matrix R, $a = Y(\text{target})$ represents the nucleotide type of the target leaf, t_n represents the edge length between node n and its parent $\text{Pa}(n)$, $p(j|i,t) = \left[e^{tR}\right]_{ji}$ represents the transition matrix of a continuous-time Markov process specified by rate matrix R, $\mathcal{D}(n)$ represents descendant leaves of node n, $Y(\mathcal{D}(n))$ represents the partial alignment column that consists of nucleotides of leaves $\mathcal{D}(n)$, $\alpha(n,i)$ represents the probability of emitting nucleotides $Y(\mathcal{D}(n))$ given the state of node n is fixed to i, and $\gamma(n,i)$ represents the probability of emitting $Y(\mathcal{D}(n))$ given the state of parent node $\text{Pa}(n)$ of n is fixed to i. As compared to the corresponding formulas for t_{MRS}, we used an extra type of dynamic programming variable $\tau_D(c_k,i) = 2\bar{t}s_k\alpha_D(c_k,i)$, which represents the product of the edge length $\bar{t}s_k$ up to concestor c_k and the probability $\alpha_D(c_k,i)$ with no substitution along the target lineage. By carefully separating the functions that depend on the integration variable s and contributions from other part of the tree, we obtain Algorithm 1 for computing $\mathbb{E}\left(T^2_{\text{MRS}}\right)$,

Algorithm 1 Compute $\mathbb{E}\left(T^2_{\text{MRS}}\right)$

compute inside and outside variables $\alpha(n,i)$, $\beta(n,i)$ for all nodes n and nucleotides $i \in \text{Nuc}$
$\alpha_D(c_0,i) \leftarrow \delta_{i,Y(c_0)}$ for $i \in \text{Nuc}$
$\tau_D(c_0,i) \leftarrow 0$ for $i \in \text{Nuc}$
$u \leftarrow 0$
for $k = 1$ to M **do**
  $\alpha_D(c_k,i) \leftarrow \sum_j \alpha_D(c_{k-1},j)p_D(j|i,t_{c_{k-1}})$
  $\tau_D(c_k,i) \leftarrow \sum_j \tau_D(c_{k-1},j)p_D(j|i,t_{c_{k-1}}) + 2\bar{t}s_{k-1,k}\alpha_D(c_k,i)$
  $u \leftarrow u + \sum_{ij} \tau_D(c_{k-1},i)\kappa(i,j)\gamma(b_k,j)\beta(c_{k-1},j)$
  $u \leftarrow u + \sum_{ij} \alpha_D(c_{k-1},i)\kappa'(i,j)\gamma(b_k,j)\beta(c_{k-1},j)$
end for
$u \leftarrow u/\mathcal{Z}(Y)$
return u

In Algorithm 1, $p_D(j|i,t) = [\exp(tR_D)]_{ji}$ represents the probability of transition $j \leftarrow i$ after time interval t without any substitution. Further, $\kappa(i,j)$ and

$\kappa'(i,j)$ are given by

$$\kappa(i,j) = \bar{t} \sum_l U_{il} U^{-1}{}_{lj} \mathcal{K}(s_{k-1,k}\bar{t}R_{Dii}, s_{k-1,k}\bar{t}\lambda_l)$$

$$\kappa'(i,j) = 2\bar{t}^2 \sum_l U_{il} U^{-1}{}_{lj} \mathcal{K}'(s_{k-1,k}\bar{t}R_{Dii}, s_{k-1,k}\bar{t}\lambda_l)$$

$$\mathcal{K}(x,y) = \begin{cases} \frac{e^x - e^y}{x-y} & \text{if } x \neq y \\ e^x & \text{if } x = y \end{cases}$$

$$\mathcal{K}'(x,y) = \frac{\partial \mathcal{K}(x,y)}{\partial x}.$$

Here, $R = U\Lambda U^{-1}$ ($\Lambda = \mathrm{diag}(\lambda_1, \ldots, \lambda_4)$) is an eigenvalue decomposition of rate matrix R.

The probability of no substitution along the target lineage is given by

$$q = \frac{\sum_i \alpha_D(c_M, i)\pi_i}{\mathcal{Z}(Y)},$$

where, c_M is the root node.

3 Inside and Outside Algorithms

The inside algorithm [3] is defined in Algorithm 2.

Algorithm 2 Compute inside variables $\alpha(n,i)$

for each leaf node n **do**
  if $y_n \in \mathrm{Nuc}$ **then**
    $\alpha(n,i) \leftarrow \delta_{i,y_n}$
  else
    $\alpha(n,i) \leftarrow 1$ for all $i \in \mathrm{Nuc}$
  end if
end for
for each internal node n (including root) in postorder traversal **do**
  $\alpha(n,i) \leftarrow \prod_{n' \in \mathrm{Ch}(n)} \sum_j \alpha(n',j) p(j|i, t_{n'})$
end for

In Algorithm 2, $\mathrm{Ch}(n)$ represents the set of child nodes of node n. δ_{ij} represents the Kronecker's symbol and assumes value 1 if $i = j$ and 0 otherwise. $p(i|j,t) = \left[e^{tR}\right]_{ij}$ represents the transition matrix of continuous time Markov processes specified by rate matrix R.

The outside variables $\beta(n,i)$ are computed by using the inside variables (Algorithm 3).

In Algorithm 2, $\mathrm{Pa}(n)$ represents the parent node of node n. $\mathrm{Sib}(n)$ represents the set of sibling nodes of node n, which have a common parent $\mathrm{Pa}(n)$. π

Algorithm 3 Compute outside variables $\beta(n, i)$

$\beta(\text{root}, i) = \pi_i$
for each node n other than root in preorder traversal **do**
  $\beta(n, i) \leftarrow \sum_j p(i|j, t_{\text{Pa}(n)})\beta(\text{Pa}(n), j)$
  $\beta(n, i) \leftarrow \beta(n, i) \prod_{n' \in \text{Sib}(n)} \sum_j \alpha(n', j)p(j|i, t_{n'})$
end for

represents the equilibrium probability distribution of the Markov process specified by rate matrix R, which can be computed from the right eigenvector of R with eigenvalue zero.

4 Optimization of Model Parameters

We optimize rate parameters and the edge lengths of the phylogenetic tree with fixed tree topology by using the maximum likelihood method. We use the LBGSB gradient descent package to maximize the likelihood [8]. The likelihood of an alignment column Y is given by,

$$\mathcal{Z}(Y) = \sum_i \alpha(\text{root}, i)\pi_i$$

which can be computed using Algorithm 2. Kiryu [5] showed that the gradient of the average log likelihood $l(\theta; D)$ ($D = \{Y_d\}$, $\theta = (\{R_{ij}\}, \{t_n\})$) can be computed as follows

$$\frac{\partial l}{\partial \theta_k} = \sum_{n,i} \frac{\partial (t_n R_{ii})}{\partial \theta_k} F_d^{(n)}(i) + \sum_{i \neq j} \frac{\partial \log(t_n R_{ij})}{\partial \theta_k} N_s^{(n)}(i, j) + \sum_i \frac{\partial \log \pi_i}{\partial \theta_k} \nu(i),$$

where θ_k is an element of parameter vector θ. $F_d^{(n)}(i)$ represents the averaged fractional duration that the state remained i along edge $(n, \text{Pa}(n))$. $N_s^{(n)}(i, j)$ represents the averaged count of transition $i \leftarrow j$ in edge $(n, \text{Pa}(n))$. $\nu(i)$ represents the averaged number of columns that have i as the state at root node [5]. For a single alignment column Y, $F_d^{(n)}(i)$, $N_s^{(n)}(i, j)$, and $\nu(i)$ are computed by

the following equations [5],

$$
\begin{aligned}
F_d^{(n)}(i) &= \frac{1}{\mathcal{Z}(Y)} \sum_{ab} \alpha(n,a) \kappa_F^{(n)}(a,b,i) \beta(n,b) \\
N_s^{(n)}(i,j) &= \frac{1}{\mathcal{Z}(Y)} \sum_{ab} \alpha(n,a) \kappa_N^{(n)}(a,b,i,j) \beta(n,b) \\
\nu(i) &= \frac{1}{\mathcal{Z}(Y)} \alpha(\mathrm{root}, i) \pi_i \\
\kappa_F^{(n)}(a,b,i) &= \sum_{kl} U_{ak} {U^{-1}}_{ki} U_{il} {U^{-1}}_{lb} \mathcal{K}(t_n \lambda_k, t_n \lambda_l) \\
\kappa_N^{(n)}(a,b,i,j) &= \sum_{kl} U_{ak} {U^{-1}}_{ki} t_n R_{ij} U_{jl} {U^{-1}}_{lb} \mathcal{K}(t_n \lambda_k, t_n \lambda_l).
\end{aligned}
$$

For multiple alignment columns, we average $F_d^{(n)}(i)$, $N_s^{(n)}(i,j)$, and $\nu(i)$ for single columns from all alignment columns.

The equilibrium distribution π is the right eigenvector of rate matrix R with eigenvalue zero. If there are no duplications at eigenvalue 0 and all off-diagonal elements $\{R_{ij} | i \neq j\}$ are independent variables, then the partial derivative of π with respect to R_{ij} $(i \neq j)$ is given by [4],

$$
\begin{aligned}
\frac{\partial \pi_k}{\partial R_{ij}} &= -(R_{ki}^{+} - R_{kj}^{+}) \pi_j \\
R^{+} &= U \Lambda^{+} U^{-1}, \Lambda^{+} = \mathrm{diag}(\lambda_1^{+}, \ldots, \lambda_4^{+}) \\
\lambda_i^{+} &= \begin{cases} \frac{1}{\lambda_i} & \text{if } \lambda_i \neq 0 \\ 0 & \text{if } \lambda_i = 0 \end{cases},
\end{aligned}
$$

where R^{+} is the Moore-Penrose pseudoinverse of rate matrix $R = U \Lambda U^{-1}$ [2]. When there is constraint among the elements of R such as in the case of the strand symmetric rate matrix, we take the sum of the gradients for related matrix elements using the partial derivative chain rule.

5 Posterior sampling of substitution history

In order to check the correctness of our algorithm numerically, we generate substitution histories on the phylogenetic tree given a multiple alignment column. For each alignment column Y, we first sample a root state from the posterior distribution,

$$
\mathbb{P}(X_{\mathrm{root}} = i | Y) = \frac{1}{\mathcal{Z}(Y)} \alpha(\mathrm{root}, i) \pi_i = \nu(i).
$$

Then, we sample sequences of states along tree edges in preorder traversal. For each edge $(n, \mathrm{Pa}(n))$, we divide the edge into K intervals, and sample the states

at the boundaries of the intervals sequentially from parent node $\mathrm{Pa}(n)$ to node n according to the conditional probability distribution

$$\begin{aligned}\mathbb{P}(X_{n,k} = i | X_{n,k-1} = j, Y) = \frac{1}{Z} \sum_a \alpha(n,a) p(a|i, (t_n(K-k)/K)) p(i|j, (t_n/K)) \\ Z = \sum_i \sum_a \alpha(n,a) p(a|i, (t_n(K-k)/K)) p(i|j, (t_n/K)). \\ k = 1, \ldots, K\end{aligned}$$

6 Forward sampling of substitution history

In order to compare our t_{MRS} measure with other indicators of conservation and variation, we simulated substitution history and alignment column using the trained model of phylogenetic tree and rate matrix. We first sample the state at the root node from the equilibrium distribution π. Then we sample state at each discretized position from the root to leaves using short time transition probability matrix $p(j|i, \Delta t) = [\exp(\Delta t R)]_{ji}$. Then the states $\{X_{n,M}\}$ at the leaf

Algorithm 4 Forward sampling of substitution history

```
for each node n in preorder traversal do
  if n is root then
    sample X_{n,K} ~ π
  else
    for k = 1 to K do
      sample X_{n,k} ~ p(j|X_{n,k-1}, (t_n/K)) where X_{n,0} = X_{Pa(n),K}
    end for
  end if
end for
return {X_{n,k}}
```

nodes $\{n \in \mathcal{L}\}$ give an alignment column. The true t_{MRS} and probability of no mutation $q \in \{0, 1\}$ in this simulation are computed by traversing substitution history $\{X_{n,k}\}$ from leaf to root along the target lineage. To imitate gap patterns of real alignments, we sampled alignments from real multiz100way dataset and masked bases with ambiguous characters at the same positions that have gap or ambiguous characters in the real dataset.

7 Computation of the Transition–Transversion Rate Ratio

The transition–transversion rate ratio r was computed as follows:

$$r = \frac{\frac{1}{|\mathrm{Transitions}|} \sum_{(ij) \in \mathrm{Transitions}} R_{ij} \pi_j}{\frac{1}{|\mathrm{Transversions}|} \sum_{(ij) \in \mathrm{Transversions}} R_{ij} \pi_j},$$

Table 1: Count of Differentially-Expressed Transcribed Enhancers with Concestor Intervals. We show the number of differentially-expressed transcribed enhancers that correspond to a specific site within the concestor interval.

Concestor Interval	Enhancer Count
Homo–Hominoidea	23232
Homo–Euarchontoglires	27767
Homo–Eutheria	14318
Homo–Mammalia	16380
Homo–Amniota	208
Homo–Tetrapoda	1438
Homo–Vertebrata	2341
Hominoidea–Euarchontoglires	25380
Hominoidea–Eutheria	26261
Hominoidea–Mammalia	22183
Hominoidea–Amniota	2724
Hominoidea–Tetrapoda	2733
Hominoidea–Vertebrata	2141
Euarchontoglires–Mammalia	539
Euarchontoglires–Tetrapoda	38
Euarchontoglires–Vertebrata	589
Euarchontoglires–Amniota	30
Eutheria–Tetrapoda	188
Eutheria–Vertebrata	750

where Transitions and Transversions are the set of transitions and transversions, respectively.

8 Tissue-Specific Transcribed Enhancers

For testing the correlation between tissue-specific enhancer expressions and concestor intervals of last mutation events, we used the differentially-expressed transcribed enhancers of Transcribed Enhancer Atlas [1] and counted the enhancers that have sites corresponding to specific concestor intervals (Table 1). Some of the differentially-expressed enhancers are annotated with UBERON anatomy ontology [6], which indicates the enhancer are differentially expressed in the annotated tissue. The number of enhancers that are differentially expressed for each UBERON term is shown in Table 2. In the significance test, we assumed the null hypothesis that enhancers containing sites with specific concestor intervals are distributed independently of UBERON tissue annotations and used the hypergeometric p-test. Since the degrees of freedom were rather large, the phyper() function in the R programming environment returned unreasonable zero p-values for some obviously insignificant hypothesis tests. Therefore, we computed Z-scores from the hypergeometric distribution and ranked data points

Table 2: Transcribed Enhancers with UBERON IDs. We show the number of transcribed enhancers that are differentially expressed in each tissue.

UBERON ID	Tissue	Enhancer Count
UBERON:0000029	lymph node	30
UBERON:0000059	large intestine	209
UBERON:0000178	blood	1335
UBERON:0000341	throat	125
UBERON:0000473	testis	643
UBERON:0000945	stomach	20
UBERON:0000948	heart	295
UBERON:0000955	brain	639
UBERON:0000970	eye	157
UBERON:0000989	penis	21
UBERON:0000992	female gonad	90
UBERON:0000995	uterus	157
UBERON:0000996	vagina	62
UBERON:0001013	adipose tissue	108
UBERON:0001043	esophagus	134
UBERON:0001044	salivary gland	59
UBERON:0001134	skeletal muscle tissue	95
UBERON:0001135	smooth muscle tissue	66
UBERON:0001255	urinary bladder	105
UBERON:0001264	pancreas	35
UBERON:0001723	tongue	133
UBERON:0001736	submandibular gland	38
UBERON:0001831	parotid gland	26
UBERON:0001981	blood vessel	158
UBERON:0001987	placenta	92
UBERON:0002046	thyroid gland	142
UBERON:0002048	lung	397
UBERON:0002097	skin of body	20
UBERON:0002106	spleen	277
UBERON:0002107	liver	84
UBERON:0002108	small intestine	143
UBERON:0002110	gallbladder	81
UBERON:0002113	kidney	190
UBERON:0002240	spinal cord	42
UBERON:0002331	umbilical cord	10
UBERON:0002360	meninx	97
UBERON:0002367	prostate gland	115
UBERON:0002370	thymus	347
UBERON:0002372	tonsil	146
UBERON:0003112	olfactory region	11
UBERON:0004054	internal male genitalia	168

Table 3: Count of Tissue Specific Genes with Concestor Intervals. We show the number of differentially expressed genes which have sites with given concestor intervals.

Concestor Interval	Gene Count
Homo-Hominoidea	4773
Homo-Euarchontoglires	4762
Homo-Eutheria	3607
Homo-Mammalia	3882
Homo-Amniota	28
Homo-Tetrapoda	1
Hominoidea-Amniota	428
Hominoidea-Euarchontoglires	4750
Hominoidea-Eutheria	4747
Hominoidea-Mammalia	4759
Hominoidea-Tetrapoda	9
Euarchontoglires-Eutheria	2531
Euarchontoglires-Mammalia	3383
Euarchontoglires-Amniota	559
Euarchontoglires-Tetrapoda	17
Eutheria-Mammalia	4333
Eutheria-Amniota	3752
Eutheria-Tetrapoda	3040
Eutheria-Vertebrata	116
Mammalia-Tetrapoda	3
Mammalia-Vertebrata	52

according to these statistics to identify significant tissues in the main text.

9 Correlation of Concestor Intervals with Tissue-Specificity of Gene Expressions

We studied the correlation between the tissue-specificity and concestor intervals for genes in a similar manner as for transcribed enhancers. We used normalized gene expression profiles of 40 tissues from RefEx database[7], which are derived from FANTOM5 CAGE data[**?**]. We defined an Entrez gene is specific to tissue A if its expression in tissue A is more than 1.5 times higher than expressions in the other tissues. The number of tissue specific genes are shown in Table **??**. We randomly assigned a representative RefSeq transcript for each gene and computed t_{MRS}, σ, and q for each exonic sequence positions and computed concestor intervals with filtering threshold $q = 0.01$. Table **??** shows the number of genes which contain sites with given concestor intervals. Then, we tested if there is significant overlap between the set of genes expressed in a specific tissue and

Table 4: Count of Genes with Tissue specific expression. We show the number of genes with tissue-specific expression for each tissue.

Tissue	Gene Count
Adipose	43
Artery Aorta	141
Bladder	14
Brain Stem	5
Breast	54
Cerebrum	271
Colon	28
Corpus Callosum Glia	116
Esophagus	181
Heart	47
Intestine	232
Kidney	116
Liver Hepato	423
Lung	59
Lymphnode	6
Muscle	310
Ovary	36
Pancreas	173
Pineal Gland	320
Pituitary	106
Placenta	272
Prostate	23
Salivary	125
Spine	34
Spleen	111
Testis	1120
Thymus	325
Thyroid Parathyroid	51
Uterus	30
Vein	5

that of genes which contain a specific concestor interval with a hypergeometric test.

References

[1] Fantom5 human enhancer tracks.

[2] A. Ben-Israel and T.N. Greville. *Generalized Inverses.* Springer, New York, USA, 2003.

[3] J. Felsenstein. Evolutionary trees from DNA sequences: a maximum likelihood approach. *J. Mol. Evol.*, 17(6):368–376, 1981.

[4] D.A. Harville. *Matrix Algebra From a Statistician's Perspective.* Springer, New York, USA, 1997.

[5] H. Kiryu. Sufficient statistics and expectation maximization algorithms in phylogenetic tree models. *Bioinformatics*, 27(17):2346–2353, Sep 2011.

[6] C. J. Mungall, C. Torniai, G. V. Gkoutos, S. E. Lewis, and M. A. Haendel. Uberon, an integrative multi-species anatomy ontology. *Genome Biol.*, 13(1):R5, Jan 2012.

[7] H. Ono, O. Ogasawara, K. Okubo, and H. Bono. RefEx, a reference gene expression dataset as a web tool for the functional analysis of genes. *Sci Data*, 4:170105, 08 2017.

[8] C. Zhu, R.H. Byrd, and J. Norcedal. L-BFGS-B: Algorithm 778: L-BFGS-B, FORTRAN routines for large scale bound constrained optimization. *ACM Transactions on Mathematical Software*, 23(4):550–560, 1997.
